# Supplementary material for: Centrioles generate a local pulse of Polo/PLK1 activity to initiate mitotic centrosome assembly
Source: EMBO J. 2022 May 3;41(11):e110891. doi: 10.15252/embj.2022110891 (PMC9156973; doi:10.15252/embj.2022110891)
Supplement: Supplementary file 1 — Appendix [file EMBJ-41-e110891-s003.pdf]

Table of contents

Appendix Supplementary Methods for Mathematical Modelling…………………………P.2-13

Appendix Tables S1-S5…………………………………………………P.14-17

Appendix References……………………………………………………P.18-19

Appendix Supplementary Methods for Mathematical Modelling

Mathematical model of PCM scaffold assembly kinetics *(Model 1)*

We assume that centriolar Spd-2 receptors, R_S, are able to convert cytoplasmic Spd-2, S, into an unstable Spd-2 scaffold, S^*, via the complex $\bar{R}_S$. The on, off, and catalytic conversion rates of this process are k_S^{on}, k_S^{off}, and $k_S^{\text{cat}} P^*/n$, respectively, where $P^*(t)$ is the total amount of active Polo at time t and $n(t)$ is the number of centrioles in the embryo at time t (which will double after every cycle), so that P^*/n describes the amount of active Polo at each centriole. In the first instance, we do not attempt to model $P^*(t)$ but instead treat it as a given function which we use as an external stimulus for the system. Although S^* is unstable, it can recruit cytoplasmic Cnn, C, and cytoplasmic Polo, P, to form the more stable complex $\bar{S}$, which can phosphorylate C to convert it into a stable Cnn scaffold form C^*. The on, off, and catalytic conversion rates of this process are k_C^{on}, k_C^{off}, and k_C^{cat}, respectively. The two scaffold forms of Spd-2 have disassembly rates $k_{S^*}^{\text{dis}}$ and $k_{\bar{S}}^{\text{dis}}$, respectively, while the disassembly rate of the Cnn scaffold is given by $k_C^{\text{dis}} C^*/n$, as we assume that this disassembly rate is proportional to the size of the Cnn scaffold. This assumption is based on our previous observation that at the start of S-phase the old mother (OM) centrosome organises a larger Cnn scaffold than the new mother (NM), but the two scaffolds ultimately grow to the same size by the end of S-phase (Conduit *et al*, 2010). As the Cnn incorporation rate is the same at OM and NM centrosomes (Conduit *et al*, 2010; S.S.W, *unpublished observations*) we infer that the rate of loss of Cnn during S-phase must be larger at the OM, indicating that the larger the Cnn scaffold, the larger the rate of Cnn loss.

The previous description can be summarised as a system of reactions

$$R_S + S \ \overset{k_S^{\text{on}}}{\underset{k_S^{\text{off}}}{\leftrightarrow}} \ \bar{R}_S \ \xrightarrow{\frac{k_S^{\text{cat}} P^*}{n}} \ R_S + \ S^*, \tag{1}$$

$$S^* + C + P \ \overset{k_C^{\text{on}}}{\underset{k_C^{\text{off}}}{\leftrightarrow}} \ \bar{S} \ \xrightarrow{k_C^{\text{cat}}} S^* + \ C^* + P, \tag{2}$$

$$S^* \ \xrightarrow{k_{S^*}^{\text{dis}}} S, \tag{3}$$

$$\bar{S} \ \xrightarrow{k_{\bar{S}}^{\text{dis}}} S + C + P, \tag{4}$$

$$C^* \ \xrightarrow{\frac{k_C^{\text{dis}} C^*}{n}} C. \tag{5}$$

For simplicity, we assume that cytoplasmic species diffuse sufficiently fast in the embryo that we may treat these variables as spatially homogeneous, and therefore we neglect spatial effects from the model. By imposing the law of mass action, we derive the following system of four ordinary differential equations (note that the explicit dependence in the dependent variables on time has been dropped)

$$\frac{d\bar{R}_S}{dt} = k_S^{\text{on}} R_S S - \left(k_S^{\text{off}} + \frac{k_S^{\text{cat}} P^*}{n}\right) \bar{R}_S, \tag{6}$$

$$\frac{dS^*}{dt} = \frac{k_S^{\text{cat}} P^* \bar{R}_S}{n} - k_C^{\text{on}} CPS^* + \ (k_C^{\text{off}} + \ k_C^{\text{cat}})\bar{S} - k_{S^*}^{\text{dis}} S^*, \tag{7}$$

$$\frac{d\bar{S}}{dt} = k_C^{\text{on}} CPS^* - \ (k_C^{\text{off}} + \ k_C^{\text{cat}})\bar{S} - \ k_{\bar{S}}^{\text{dis}} \bar{S}, \tag{8}$$

$$\frac{dC^*}{dt} = k_C^{\text{cat}} \bar{S} - \frac{k_C^{\text{dis}} C^{*2}}{n}, \tag{9}$$

where the PCM quantities, $S^*, \bar{S}, C^*, P^*, R_S$ and $\bar{R}_S$ are defined as the total number of the corresponding species in the embryo (i.e. dimensionless units), and the cytoplasmic quantities, $S, C,$ and P, are defined as the volumetric concentration of the corresponding species (i.e. units m^{-3}). We assume, for simplicity, that the embryo is a closed system which implies that the total amount Spd-2 (S_0) and Cnn (C_0) in the embryo is conserved.

Further, since the total amount of Polo in the system (P_0) is large (Casas-Vila *et al*, 2017) we treat cytoplasmic Polo as a prescribed constant unaffected by absorption into the scaffold. Finally, we assume that the total number of Spd-2 receptors in the embryo is proportional to the number of centrioles. These constraints read

$$R_S + \bar{R}_S = {r_S}_0 n, \tag{10}$$

$$\bar{R}_S + VS + S^* + \bar{S} = S_0, \tag{11}$$

$$VC + C^* + \bar{S} = C_0, \tag{12}$$

$$VP = P_0, \tag{13}$$

where ${r_S}_0$ is the total number of receptors per centriole and V is the volume of the embryo.

These equations describe the total amount of each species in the embryo. However, it is useful to describe the model on a per-centriole basis. We do this by defining the auxiliary (lower case) per-centriole variables: $R_S = nr_S$, $\bar{R}_S = n\bar{r}_S$, $S^* = ns^*$, $\bar{S} = n\bar{s}$, $C^* = nc^*$, and $P^* = np^*$. In terms of these variables, our system reads

$$\frac{d\bar{r}_S}{dt} = k_S^{\text{on}} r_S S - \left(k_S^{\text{off}} + k_S^{\text{cat}} p^*\right)\bar{r}_S - \frac{\bar{r}_S}{n}\frac{dn}{dt}, \tag{14}$$

$$\frac{ds^*}{dt} = k_S^{\text{cat}} p^* \bar{r}_S - \frac{k_C^{\text{on}} C P_0 s^*}{V} + (k_C^{\text{off}} + k_C^{\text{cat}})\bar{s} - k_{S^*}^{\text{dis}} s^* - \frac{s^*}{n}\frac{dn}{dt}, \tag{15}$$

$$\frac{d\bar{s}}{dt} = \frac{k_C^{\text{on}} C P_0 s^*}{V} - (k_C^{\text{off}} + k_C^{\text{cat}})\bar{s} - k_{\bar{S}}^{\text{dis}} \bar{s} - \frac{\bar{s}}{n}\frac{dn}{dt}, \tag{16}$$

$$\frac{dc^*}{dt} = k_C^{\text{cat}} \bar{s} - k_C^{\text{dis}} c^{*2} - \frac{c^*}{n}\frac{dn}{dt}, \tag{17}$$

subject to

$$r_S + \bar{r}_S = {r_S}_0, \tag{18}$$

$$VS + n(\bar{r}_S + s^* + \bar{s}) = S_0, \tag{19}$$

$$VC + n(c^* + \bar{s}) = C_0. \tag{20}$$

While equations (14) – (20), subject to the appropriate initial conditions, are sufficient to describe the system, it is convenient for its mathematical analysis to instead formulate the model in terms of “dimensionless” variables. Through this process, we determine the dimensionless parameter groups (e.g. the ratio of the reaction rates to the cell cycle timescale) which govern the dynamics of the system, which in turn enables us to simplify the system and reduce the number of independent variables in the model. We non-dimensionalise the system by using the following scalings

$$r_S, \bar{r}_S, s^*, \bar{s}, c^* \sim r_{S_0}, \qquad S \sim \frac{S_0}{V}, \qquad C \sim \frac{C_0}{V}, \qquad p^* \sim p_{\mathrm{max}}, \qquad t \sim T, \tag{21}$$

where T is the typical period of the cell cycle, and p_{max} is the maximum amplitude of the imposed Polo activity. In terms of dimensionless variables, the model reads

$$\frac{d\bar{r}_S}{dt} = K_S^{\mathrm{on}} r_S S - \left(K_S^{\mathrm{off}} + K_S^{\mathrm{cat}} p^*\right)\bar{r}_S - \frac{\bar{r}_S}{n}\frac{dn}{dt}, \tag{22}$$

$$\frac{ds^*}{dt} = K_S^{\mathrm{cat}} p^* \bar{r}_S - K_C^{\mathrm{on}} C s^* + (K_C^{\mathrm{off}} + K_C^{\mathrm{cat}})\bar{s} - K_{S^*}^{\mathrm{dis}} s^* - \frac{s^*}{n}\frac{dn}{dt}, \tag{23}$$

$$\frac{d\bar{s}}{dt} = K_C^{\mathrm{on}} C s^* - (K_C^{\mathrm{off}} + K_C^{\mathrm{cat}})\bar{s} - K_{\bar{S}}^{\mathrm{dis}} \bar{s} - \frac{\bar{s}}{n}\frac{dn}{dt}, \tag{24}$$

$$\frac{dc^*}{dt} = K_C^{\mathrm{cat}} \bar{s} - K_C^{\mathrm{dis}} c^{*2} - \frac{c^*}{n}\frac{dn}{dt}, \tag{25}$$

subject to

$$r_S + \bar{r}_S = 1, \tag{26}$$

$$S + \delta_S n(\bar{r}_S + s^* + \bar{s}) = 1, \tag{27}$$

$$C + \delta_C n(c^* + \bar{s}) = 1, \tag{28}$$

where

$$K_S^{\mathrm{on}} = k_S^{\mathrm{on}} T S_0/V, \quad K_S^{\mathrm{off}} = k_S^{\mathrm{off}} T, \quad K_S^{\mathrm{cat}} = k_S^{\mathrm{cat}} p_{\mathrm{max}} T, \quad K_{S^*}^{\mathrm{dis}} = k_{S^*}^{\mathrm{dis}} T, \quad K_{\bar{S}}^{\mathrm{dis}} = k_{\bar{S}}^{\mathrm{dis}} T,$$

$$K_C^{\text{on}} = k_C^{\text{on}} T C_0 P_0 / V^2, \qquad K_C^{\text{off}} = k_C^{\text{off}} T, \qquad K_C^{\text{cat}} = k_C^{\text{on}} T, \qquad K_C^{\text{dis}} = k_C^{\text{dis}} r_{S_0} T,$$

$$\delta_S = \frac{r_{S_0}}{S_0}, \qquad \delta_C = \frac{r_{S_0}}{C_0}, \qquad \delta_{p^*} = \frac{r_{S_0}}{p_{\text{max}}}. \tag{29}$$

Given a solution to this system, the total size of the Spd-2 and Cnn scaffolds and total amount of active Polo surrounding each centriole are given by

$$S_{\text{tot}} = \bar{r}_S + s^* + \bar{s}, \tag{30}$$

$$C_{\text{tot}} = c^* + \bar{s}, \tag{31}$$

$$P_{\text{tot}} = p^* + \delta_{p^*} \bar{s}, \tag{32}$$

where S_{tot}, C_{tot}, and P_{tot} are dimensionally scaled with S_0, C_0, and p_{max}, respectively.

To allow us to compare accurately the output from our models to the experimental data we first determined reasonable initial conditions, as the centrosomes in our experiments are already initially associated with some PCM (that was acquired in the previous cycle) at the start of S-phase. To do this, we first solve (22) – (28) subject to the initial conditions $\bar{r}_S = s^* = \bar{s} = c^* = 0$. (i.e. no Spd-2 or Cnn scaffold is assembled around the centriole). Since the system is approximately cyclic, we then use the final values output by this solution, $\bar{r}_S = \bar{r}_{S_0}$, $s^* = s_0^*$, $\bar{s} = \bar{s}_0$, as our new initial values. Since the Cnn scaffold divides and partially breaks away during centriole separation, we cannot impose the cyclic condition on Cnn. However, since the output of c^* is $O(1)$, this suggests that an $O(1)$ input is consistent with our model and therefore we set $c^* = 1$ as our initial condition. In this way, the centrioles in our model start the cycle already associated with some Spd-2 and Cnn scaffold that they acquired in the previous cycle, as is the case with our experimental data.

In Figure 2B, we plot the incorporation of Spd-2 and Cnn into the PCM, S_{tot} and C_{tot}, over the duration of a single cycle by solving (22) – (28) subject to the initial conditions $\bar{r}_S = \bar{r}_{S_0}$, $s^* = s_0^*$, $\bar{s} = \bar{s}_0$, $c^* = 1$, the parameter values given in **Appendix Table S4**, and the constraint that the number of centrioles is constant during the cycle, $n \equiv 1$ without loss of generality. We also plot the prescribed Polo activity (i.e. the oscillation in $p^*(t)$ that we impose on the system), $p^*(t) \coloneqq \frac{1}{2}(1 - \cos(2\,\pi\,t))$, and the total Polo at the centriole, P_{tot}. The amplitudes in all the solutions have been normalised to 1.

The initial conditions and parameters used in this model are listed in Appendix Table S4. Our justification for choosing these parameter values is presented in a later Section.

-

We note that, in our model, the dissociation rates of the C^* and $\bar{S}$ scaffolds have different functional forms. To investigate if this contributes to the different behaviour of the C^* and $\bar{S}$ scaffolds we compare in the graphs below the model output in the case in which the exponent of the C^* disassembly term ($K_C^{\mathrm{dis}} {c^*}^{\alpha}$) is varied, and the case in which the disassembly rate itself is varied. This shows that C^* behaviour is primarily determined by the order of magnitude of the disassembly rate, rather than its exponent.

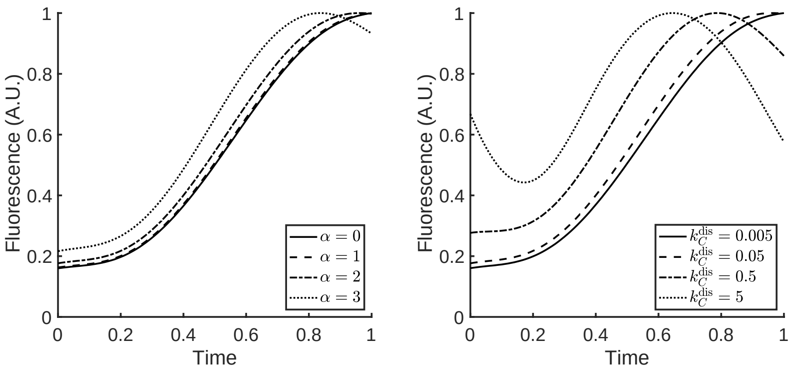

<u>Mathematical model of centriolar Polo activity *(Model 2)*</u>

Model 1 assumed a given oscillation in Polo. Next, we describe a model for how such an oscillation in Polo activity might be generated by the centriole through the interaction between Polo and its receptors at the centriole surface, such as Ana1 (Alvarez-Rodrigo et al., 2021). We assume that these receptors, R_P^{off}, are initially inactive and unable to bind Polo. To initiate mitotic PCM assembly, the receptors are activated at a rate k_R^{on} due to their phosphorylation by a protein kinase, which is most likely a Cdk/Cyclin, or a kinase that is regulated by the Cdk/Cyclins (such as Polo or Aurora A). This new form, which we denote R_P, is able to bind Polo with on and off rates k_P^{on} and k_P^{off}, respectively, to form the complex $\bar{R}_P$. We assume that the Polo in this complex is active and able to initiate mitotic PCM assembly as described by Model 1. We also assume that this active form of Polo instigates the deactivation of the receptors at a rate $k_R^{\text{off}}P^*/n$. This final form, which we denote $\bar{R}_P^{\text{off}}$, is unable to bind or activate Polo. This system likely resets itself between cycles when $\bar{R}_P^{\text{off}}$ is dephosphorylated to regenerate R_P^{off}, but we do not model this reset here. Finally, we assume that the reactions occurring in the PCM are the same as before, with the active centriolar Polo (in this instance given by $P^* \equiv \bar{R}_P$) now forming part of the solution to our model. The reactions describing the generation of Polo read

$$R_P^{\text{off}} \rightarrow^{k_R^{\text{on}}} R_P, \tag{33}$$

$$R_P + P \leftrightarrow^{k_P^{\text{on}}}_{k_P^{\text{off}}} \bar{R}_P, \tag{34}$$

$$R_P \rightarrow^{\frac{k_R^{\text{off}} P^*}{n}} \bar{R}_P^{\text{off}}, \tag{35}$$

By imposing the law of mass action, we obtain the following system of ODEs,

$$\frac{dR_P^{\text{off}}}{dt} = -k_R^{\text{on}} R_P^{\text{off}}, \tag{36}$$

$$\frac{dR_P}{dt} = k_R^{\text{on}} R_P^{\text{off}} - k_P^{\text{on}} P R_P + k_P^{\text{off}} \bar{R}_P - \frac{k_R^{\text{off}} P^*}{n} R_P, \tag{37}$$

$$\frac{d\bar{R}_P}{dt} = k_P^{\text{on}} P R_P - k_P^{\text{off}} \bar{R}_P, \tag{38}$$

$$\frac{d\bar{R}_P^{\text{off}}}{dt} = \frac{k_R^{\text{off}} P^*}{n} R_P, \tag{39}$$

$$P^* = \bar{R}_P, \tag{40}$$

We also assume that the total number of Polo receptors at each centriole, ${r_P}_0$, is conserved, which reads

$$R_P^{\text{off}} + R_P + \bar{R}_P + \bar{R}_P^{\text{off}} = n{r_P}_0. \tag{41}$$

As before, we write the system in per-centriole variables, and non-dimensionalise by setting $R_P^{\text{off}} = n{r_P}_0 r_P^{\text{off}}$, $R_P = n{r_P}_0 r_P$, $\bar{R}_P = n\bar{R}_P {r_P}_0$, and $\bar{R}_P^{\text{off}} = n{r_P}_0 \bar{r}_P^{\text{off}}$, and $P^* = n{r_P}_0 p^*$ so that the dimensionless model reads

$$\frac{dr_P^{\text{off}}}{dt} = -K_R^{\text{on}} r_P^{\text{off}}, \tag{42}$$

$$\frac{dr_P}{dt} = K_R^{\text{on}} r_P^{\text{off}} - K_P^{\text{on}} r_P + K_P^{\text{off}} \bar{r}_P - K_R^{\text{off}} p^* r_P, \tag{43}$$

$$\frac{d\bar{r}_P}{dt} = K_P^{\text{on}} r_P - K_P^{\text{off}} \bar{r}_P, \tag{44}$$

$$\frac{d\bar{r}_P^{\text{off}}}{dt} = K_R^{\text{off}} p^* r_P, \tag{45}$$

$$p^* = \bar{r}_P, \tag{46}$$

subject to

$$r_P^{\text{off}} + r_P + \bar{r}_P + \bar{r}_P^{\text{off}} = 1, \tag{47}$$

where

$$K_P^{\text{on}} = k_P^{\text{on}} P_0 T, \qquad K_P^{\text{off}} = k_P^{\text{off}} T, \qquad K_R^{\text{on}} = k_R^{\text{on}} T, \qquad K_R^{\text{off}} = k_R^{\text{off}} r_{P_0} T. \tag{48}$$

Note that we have scaled P^* with r_{P_0} in this instance rather than p_{max} since the maximum amplitude of the Polo activity is not known *a priori.,* and, since the receptors generate the active Polo in this model, this is the correct scaling for P^*.

In this model, the total amount of Polo in the centrosome is given by

$$P_{\text{tot}} = p^* + \delta_r \bar{s}, \tag{49}$$

where $\delta_r = \frac{r_{S_0}}{r_{P_0}}$.

As before, to determine the appropriate initial conditions, we first solve the model subject to $r_P = \bar{r}_P = \bar{r}_S = s^* = \bar{s} = c^* = 0, r_P^{\text{off}} = 1$ to compute the output $r_{P_0}, \bar{r}_{P_0}, \bar{r}_{S_0}, s_0^*, \bar{s}_0, c_0^*$. Our new initial conditions are then given by setting $r_P = r_{P_0}$, $\bar{r}_P = \bar{r}_{P_0}$, $\bar{r}_S = \bar{r}_{S_0}$, $s^* = s_0^*$, $\bar{s} = \bar{s}_0$, $r_P^{\text{off}} = 1 - r_{P_0} - \bar{r}_{P_0}$.

In Figure 4A, we plot the centriolar Polo, p^*, the total Polo, P_{tot}, the Spd-2 scaffold size, S_{tot}, and the Cnn scaffold size C_{tot}, found by solving (22) – (28) and (43) – (48) with the parameter values given in **Appendix Tables S4 and S5**. As before, to determine the appropriate initial conditions, we first solve the model subject to $r_P = \bar{r}_P = \bar{r}_S = s^* = \bar{s} =$

$c^* = 0, r_P^{\text{off}} = 1$ to compute the output ${r_P}_0, {\bar{r}_P}_0, {\bar{r}_S}_0, s_0^*, \bar{s}_0$. Our new initial conditions are then given by setting $r_P = {r_P}_0$, $\bar{r}_P = {\bar{r}_P}_0$, $\bar{r}_S = {\bar{r}_S}_0$, $s^* = s_0^*$, $\bar{s} = \bar{s}_0$, $r_P^{\text{off}} = 1 - {r_P}_0 - {\bar{r}_P}_0$, and $c^* = 1$. All solutions have been normalised.

In Figure 6, we plot the total Polo under normal conditions (parameter values given in Appendix Tables S4 and S5) as well as half dose Ana1 (${r_P}_0 \to 0.5{r_P}_0$, i.e. $\delta_r \to 2\delta_r$ and $K_R^{\text{off}} \to 0.5K_R^{\text{off}}$) and half dose Spd-2 ($S_0 \to 0.5S_0$, i.e. $\delta_S \to 2\delta_S$ and $K_S^{\text{on}} \to 0.5K_S^{\text{on}}$). All solutions have been normalised with respect to the wild type solution.

Justification of parameter values

We drew on a number of sources to estimate the relative magnitudes of the dimensionless reaction rate parameters (Appendix Tables S4 and S5) for the models depicted schematically in Figures 2B and 4A.

Due to the rapid fluorescence recovery rates observed in FRAP experiments (Conduit et al., 2010, 2014; Feng et al., 2017) (Figure 5), it follows that the Polo reaction rates, $K_P^{\text{on,off}}$, Spd-2 reaction rates, $K_S^{\text{on,off,cat}}$, and Spd-2 scaffold disassembly rate, $K_{S^*}^{\text{dis}}$, are large relative to the cell cycle timescale. Furthermore, due to the large size and rapid construction rate of the Cnn scaffold, it follows that $K_C^{\text{on,off,cat}}$, are also large. By contrast, since FRAP data shows that the fluorescence level of the Cnn scaffold fails to fully recover even over an entire nuclear cycle (Figure 5), it follows that the Cnn scaffold disassembly rate, K_C^{dis}, is small by comparison with the cell cycle timescale. In order to quantify the

modelling assumption that the $\bar{S}$ scaffold is more stable than the S^* scaffold, we prescribe that $1 \sim K_{\bar{S}}^{\text{dis}} \ll K_{S^*}^{\text{dis}}$.

We expect that the catalytic conversion, i.e. activation and subsequent release, of Spd-2 and Cnn is a more complex process than unbinding alone, so we assume the catalytic conversion rates are slower than the off rates. However, for simplicity, we assume that these rates are all of a similar order of magnitude. To ensure that the Spd-2 scaffold can convert Cnn into a scaffold before it disassembles, we also assume that the Spd-2 disassembly rate is less than the catalytic conversion rate of Cnn.

We make the additional assumption that K_P^{on} is larger than K_P^{off} since the amount of Polo in the embryo is large (Casas-Vila *et al*, 2017) and K_P^{on} is proportional to P_0. By contrast, we assume that K_S^{on} is smaller than K_S^{off} and K_S^{cat} as the amount of Spd-2 in the embryo is comparatively small (Casas-Vila *et al*, 2017) and K_S^{on} is proportional to S_0. Our own Fluorescence Correlation Spectroscopy (FCS) data indicate that Polo is present in the cytoplasm at 3-5X higher levels than Spd-2 or Cnn (Thomas Steinacker, *personal communication*).

Since the Polo receptors are required to both activate and deactivate in a single cycle, it follows that $K_R^{\text{on,off}}$ are sufficiently large (i.e. greater than order unity) that the receptors have time to reset, but not so large that the resetting is instantaneous. We therefore suppose that they are ≈ 10 for simplicity.

These assumptions may be combined to read

$$1 \ll K_P^{\text{off}} < K_P^{\text{on}} \tag{1}$$

$$1 \ll K_S^{\text{on}} < K_S^{\text{cat}} < K_S^{\text{off}} \tag{2}$$

$$1 \sim K_{\bar{S}}^{\text{dis}} \ll K_{S^*}^{\text{dis}} < K_C^{\text{cat}} \leq K_C^{\text{on}} < K_C^{\text{off}} \tag{3}$$

$$K_C^{\text{dis}} \ll 1 \tag{4}$$

$$K_R^{\text{on,off}} = O(10) \tag{5}$$

Since the total amount of Spd-2 and Cnn in the embryo likely greatly exceeds the number of Spd-2 receptors at the centriole, we prescribe $\delta_S \ll 1$. Furthermore, we observe through Fluorescence Correlation Spectroscopy (FCS) analysis that the total amount of Spd-2 and Cnn in the embryo are similar (Thomas Steinacker, *personal communication*) and therefore $\delta_S \approx \delta_C$. On the other hand, since the total amount of Polo bound to the centriole cannot exceed the number of receptors, it follows that $\delta_{P^*} > 1$. Finally, since we are unable to determine the relative sizes of r_{P_0} and r_{S_0}, we suppose that $\delta_r = O(1)$ for simplicity. Hence, these parameters satisfy

$$\delta_S = \delta_C \ll 1 \qquad \delta_{P^*} > 1 \qquad \delta_r = O(1) \tag{6}$$

The assumptions outlined above lay the foundation for the parameter values we have chosen. However, it worth noting that the characteristic behaviour of the response curves is unaltered by doubling or halving any of these values, and therefore the particular regime we analyse in this manuscript is robust to variation in the parameters.

Appendix Tables S1-S5

Appendix Table S1: *Drosophila* stocks used in this study

Allele	Source
cnn^{f04547}	Exelixis stock no. f04547, Exelixis Stock Centre (Harvard Medical School, Boston, MA).
cnn^{HK21}	(Megraw *et al*, 1999; Vaizel-Ohayon & Schejter, 1999)
Ubq-NG-Cnn	Generated by Lisa Gartenmann; appears to be fully functional and rescues $cnn^{-/-}$ mutant.
Ubq-Spd-2-GFP	(Dix & Raff, 2007)
Spd-2-NG (CRISPR)	Generated for this study; appears to be fully functional and is homozygous viable and fertile.
$Spd\text{-}2^{z35711}$	(Giansanti *et al*, 2008)
$Spd\text{-}2^{G20143}$	(Dix & Raff, 2007)
Polo-TRAP-GFP	(Buszczak *et al*, 2007); appears to not be fully functional and is only viable as a heterozygote.
Ubq-Spd-2-mCherry	(Alvarez-Rodrigo *et al*, 2019)
$ana1^{mecB}$	(Blachon *et al*, 2009; Avidor-Reiss *et al*, 2004)
Ubq-Ana1-mCherry	(Alvarez-Rodrigo *et al*, 2020)
Ubq-Ana1-S34T-mCherry	(Alvarez-Rodrigo *et al*, 2020)
Ubq-Spd-2-S16T-mCherry	(Alvarez-Rodrigo *et al*, 2019); described in this previous publication as Spd-2-CONS.

Appendix Table S2: *Drosophila* stocks used in specific experiments

Genotype	Experiments	Figure
Ubq-NG-Cnn, cnn^{f04547} / cnn^{HK21}	1) Dynamics of NG-Cnn or Polo-GFP across nuclear cycles 11 – 13<br>2) Recovery rate of NG-Cnn or Polo-GFP in early S-phase of nuclear cycles 11 – 13 by FRAP	1A, 1B, 5C, EV1, S3
Polo-TRAP-GFP / +		1A, 1B, 5A 6A, 6B, EV1
Ubq-Spd-2-GFP ; Spd-2^{z35711} / Spd-2^{G20143}	Dynamics of Spd-2-GFP across nuclear cycle 11 – 13	1A, 1B, EV1
Spd-2-NG (CRISPR)	Recovery rate of Spd-2-NG in the early S-phase of nuclear cycles 11 – 13 by FRAP	5B, S3
cnn^{f04547}, Ubq-GFP-Cnn / cnn^{HK21} ; Ubq-Spd-2-mCherry, Spd-2^{G20143} / Spd-2^{z35711}	GFP-Cnn or Polo-GFP co-expressed with Spd-2-mCherry to measure the relative timing of their dynamics	1C
Polo-TRAP-GFP / Ubq-Spd-2-mCherry		1D
Ubq-Ana1-mCherry / + ; Polo-TRAP-GFP / +	Polo-GFP in the presence of Ana1-mCherry or Spd-2-mCherry mutants that have significantly reduced number of Polo binding sites.	3A, 3B
Ubq-Ana1-S34T-mCherry / + ; Polo-TRAP-GFP / +		
Polo-TRAP-GFP / Ubq-Spd-2-mCherry, spd-2^{G20143}		3A, 3C
Polo-TRAP-GFP / Ubq-Spd-2-S16T-mCherry, spd-2^{G20143}		

Polo-TRAP-GFP / Spd-2^{z35711}	Polo-GFP with a reduced dosage of endogenous Spd-2 or Ana1	6A
Polo-TRAP-GFP / ana1^{mecB}		6B
cnn^{f04547} / cnn^{HK21}	Embryo without endogenous Cnn	EV1
Ubq-NG-Cnn, cnn^{f04547} / +	NG-Cnn or GFP-Cnn used in this study with endogenous Cnn to compare their relative level	
cnn^{f04547}, Ubq-GFP-Cnn / +		
Spd-2^{G20143} / Spd-2^{z35711}	Embryo without endogenous Spd-2	EV1
Ubq-Spd-2-GFP ; Spd-2^{z35711} / +	Spd-2-GFP or Spd-2-NG used in this study with endogenous Spd-2 to compare their relative level	
Spd-2-NG (CRISPR) / +		
SWFT	Embryos heterozygous of Spd-2 or Ana1 mutant to show that mutant gene can lower the expression of Spd-2 or Ana1	EV5
Spd-2^{z35711} / +		
ana1^{mecB} / +		

Appendix Table S3: Models used for feature extraction of the Cnn data

Centrosomal fluorescence of Cnn (Cycle 11)—Linear Increase
Equation:<br>$Y = m * X + c$<br><br>*Initial intensity:* c<br>*Maximum intensity:* $m * X_{last\_frame} - c$<br>*Growth Period:* X_{last_frame}<br>*Growth Rate:* m

Centrosomal fluorescence of Cnn (Cycle 12)—Linear Increase + Plateau
Equation:<br>$Y = m * X + c$ *when* $X < X_0$<br>$Y = b$ *when* $X > X_0$<br>*where* $X_0 > 0, b > 0$<br><br>*Initial Intensity:* c<br>*Maximum Intensity:* b<br>*Growth Period:* X_0<br>*Growth Rate:* m
Centrosomal fluorescence of Cnn (Cycle 13)—Linear Increase + Linear Decrease
Equation:<br>$Y = m_0 * X + c$ *when* $X < X_0$<br>$Y = m_1 * X + c$ *when* $X > X_0$<br>*where* $X_0 > 0, m_0 > 0, m_1 < 0$<br><br>*Initial Intensity:* c<br>*Maximum Intensity:* $m_0 * X_0 + c$<br>*Growth Period:* X_0<br>*Growth Rate:* m_0<br>*Decrease Rate:* m_1

Appendix Table S4: Initial Conditions and Parameters used in Model 1

K_S^{on}	K_S^{off}	K_S^{cat}	$K_{S^*}^{\mathrm{dis}}$	$K_{\bar{S}}^{\mathrm{dis}}$	K_C^{on}	K_C^{off}	K_C^{cat}	K_C^{dis}	δ_S	δ_C	δ_{p^*}
20	200	100	20	1	100	100	50	0.05	0.001	0.001	10

Appendix Table S5: Initial Conditions and Parameters used in Model 2

K_P^{on}	K_P^{off}	K_R^{on}	K_R^{off}	δ_r
100	50	10	20	5

Appendix References

Alvarez-Rodrigo I, Steinacker TL, Saurya S, Conduit PT, Baumbach J, Novak ZA, Aydogan MG, Wainman A & Raff JW (2019) Evidence that a positive feedback loop drives centrosome maturation in fly embryos. *eLife* 8

Alvarez-Rodrigo I, Wainman A & Raff JW (2020) Ana1 recruits PLK1 to mother centrioles to promote mitotic PCM assembly and centriole elongation. *bioRxiv*: 2020.08.11.244194 doi:10.1101/2020.08.11.244194 [PREPRINT]

Avidor-Reiss T, Maer AM, Koundakjian E, Polyanovsky A, Keil T, Subramaniam S & Zuker CS (2004) Decoding cilia function: Defining specialized genes required for compartmentalized cilia biogenesis. *Cell* 117: 527–539

Blachon S, Cai X, Roberts KA, Yang K, Polyanovsky A, Church A & Avidor-Reiss T (2009) A proximal centriole-like structure is present in drosophila spermatids and can serve as a model to study centriole duplication. *Genetics* 182: 133–144

Buszczak M, Paterno S, Lighthouse D, Bachman J, Planck J, Owen S, Skora AD, Nystul TG, Ohlstein B, Allen A, *et al* (2007) The carnegie protein trap library: A versatile tool for drosophila developmental studies. *Genetics* 175: 1505–1531

Casas-Vila N, Bluhm A, Sayols S, Dinges N, Dejung M, Altenhein T, Kappei D, Altenhein B, Roignant J-Y & Butter F (2017) The developmental proteome of Drosophila melanogaster. *Genome Res* 27: 1273–1285

Conduit PT, Brunk K, Dobbelaere J, Dix CI, Lucas EP & Raff JW (2010) Centrioles regulate centrosome size by controlling the rate of Cnn incorporation into the PCM. *Curr Biol* 20: 2178–2186

Dix CI & Raff JW (2007) Drosophila Spd-2 recruits PCM to the sperm centriole, but Is dispensable for centriole duplication. *Current Biology* 17: 1759–1764

Giansanti MG, Bucciarelli E, Bonaccorsi S & Gatti M (2008) Drosophila SPD-2 is an essential centriole component required for PCM recruitment and astral-microtubule nucleation. *Current Biology* 18: 303–309

Gratz SJ, Ukken FP, Rubinstein CD, Thiede G, Donohue LK, Cummings AM & O'Connor-Giles KM (2014) Highly specific and efficient CRISPR/Cas9-catalyzed homology-directed repair in Drosophila. *Genetics* 196: 961–971

Lucas EP & Raff JW (2007) Maintaining the proper connection between the centrioles and the pericentriolar matrix requires Drosophila centrosomin. *J Cell Biol* 178: 725–732

Megraw TL, Li K, Kao LR & Kaufman TC (1999) The centrosomin protein is required for centrosome assembly and function during cleavage in Drosophila. *Development* 126: 2829–2839

Novak ZA, Conduit PT, Wainman A & Raff JW (2014) Asterless licenses daughter centrioles to duplicate for the first time in Drosophila embryos. *Curr Biol* 24: 1276–1282

Port F, Chen H-M, Lee T & Bullock SL (2014) Optimized CRISPR/Cas tools for efficient germline and somatic genome engineering in Drosophila. *Proc Natl Acad Sci USA* 111: E2967-76

Port F, Muschalik N & Bullock SL (2015) Systematic Evaluation of Drosophila CRISPR Tools Reveals Safe and Robust Alternatives to Autonomous Gene Drives in Basic Research. *G3 (Bethesda)* 5: 1493–1502

Raff JW, Kellum R & Alberts B (1994) The Drosophila GAGA transcription factor is associated with specific regions of heterochromatin throughout the cell cycle. *EMBO J* 13: 5977–5983

Soille P (2004) Background Notions. In *Morphological Image Analysis: Principles and Applications*, Soille P (ed) pp 15–62. Berlin, Heidelberg: Springer

Tinevez J-Y, Perry N, Schindelin J, Hoopes GM, Reynolds GD, Laplantine E, Bednarek SY, Shorte SL & Eliceiri KW (2016) TrackMate: An open and extensible platform for single-particle tracking. *Methods* 115: 80–90

Vaizel-Ohayon D & Schejter ED (1999) Mutations in centrosomin reveal requirements for centrosomal function during early Drosophila embryogenesis. *Current Biology* 9: 889–898
