# Supplementary material for: Centrioles generate a local pulse of Polo/PLK1 activity to initiate mitotic centrosome assembly
Source: EMBO J. 2022 May 3;41(11):e110891. doi: 10.15252/embj.2022110891 (PMC9156973; doi:10.15252/embj.2022110891)
Supplement: Supplementary file 2 — Expanded View Figures PDF [file EMBJ-41-e110891-s002.pdf]

Expanded View Figures

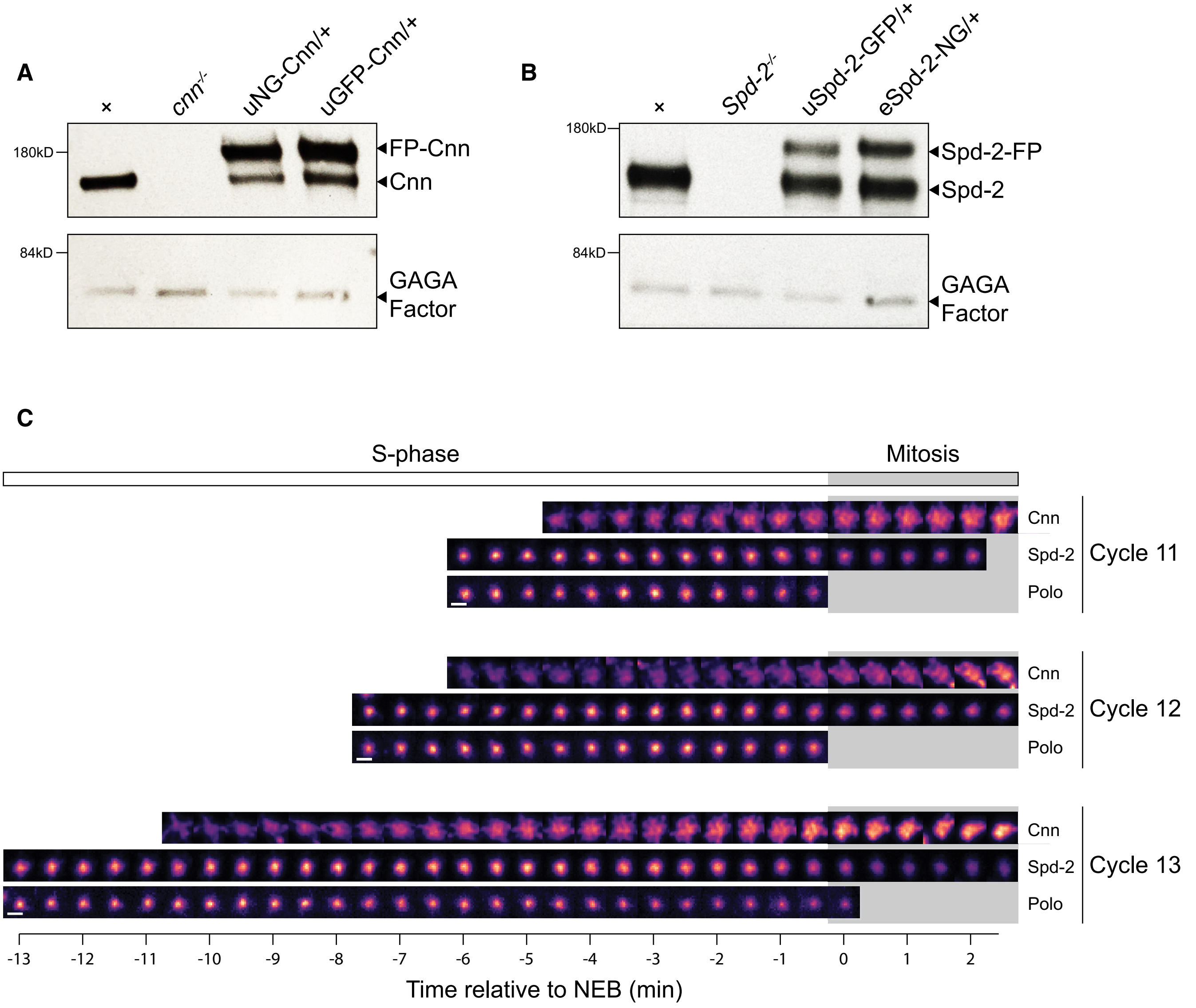

Figure EV1. Analysing the relative expression levels of the fluorescent fusion-proteins used to quantify centrosome recruitment dynamics.

A, B Western blots compare the relative expression levels in syncytial embryos of the GFP- and NG-fusion proteins (FP) used here to quantify the centrosomal recruitment dynamics of Cnn (A) or Spd-2 (B). This analysis reveals that GFP-Cnn and NG-Cnn expressed transgenically from the ubiquitin promoter (u) are present at slightly higher levels than the endogenous untagged Cnn. In contrast, Spd-2-GFP expressed transgenically from the ubiquitin promoter (u) and Spd-2-NG expressed as a CRISPR knock-in at the endogenous Spd-2 locus (e) are both present at slightly lower levels than the endogenous protein. Western blots of serial-dilutions of these samples indicate that the uNG-Cnn and uGFP-Cnn are overexpressed by ~2–3 fold compared to the endogenous protein, and that uSpd-2-GFP and eSpd-2-NG are underexpressed by ~2 fold. These blots were also probed with anti-GAGA factor antibodies as a loading control (Raff *et al*, 1994). The brightness and contrast were adjusted for optimal display.

C Images show the recruitment of either mNG-Cnn, Spd-2-GFP or Polo-GFP at an exemplar centrosome during nuclear cycles 11, 12 and 13. All images are aligned to nuclear envelope breakdown (NEB; $t = 0$). The white parts of the graphs indicate S-phase and the grey parts mitosis. The first (most leftward) image in each series is taken when the two centrosomes associated with each nucleus at the end of mitosis have first completely separated from one another in early S-phase; because the Cnn scaffold is significantly larger than the Spd-2 or Polo scaffold, it takes longer for the two centrosomes to fully separate, so there are less images of Cnn in S-phase. Scale bar = 1 μm.

© 2022 The Authors

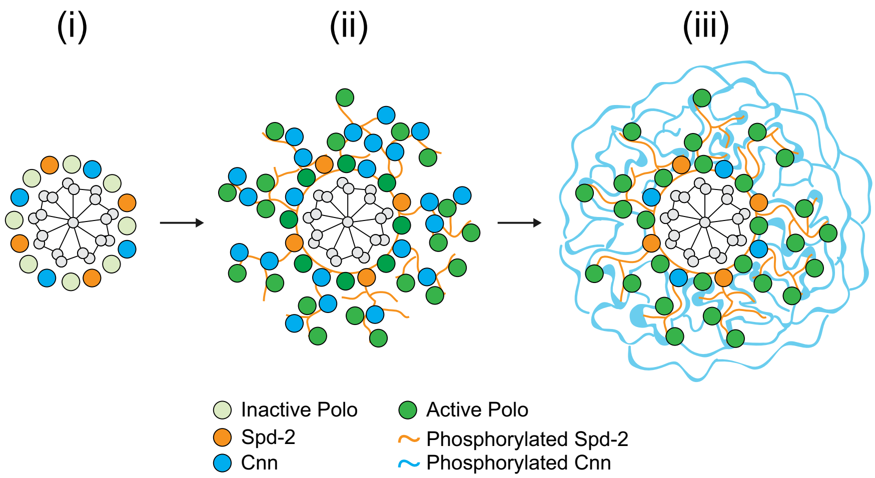

Figure EV2. A molecular model of how Spd-2, Polo and Cnn cooperate to form a mitotic PCM scaffold.

Cartoon illustrates the assembly of the Spd-2/Polo/Cnn mitotic PCM scaffold in *Drosophila*. During interphase (i), Spd-2, Polo and Cnn are recruited to a toroid that surrounds the mother centriole (Fu & Glover, 2012). Polo is presumably inactive, and Spd-2 and Cnn are presumably not phosphorylated. As cells prepare to enter mitosis (ii), Polo is activated at the centriole and the centrosomal Spd-2 becomes phosphorylated, allowing it to assemble into a scaffold that can flux outwards away from the centriole. The phosphorylated Spd-2 scaffold (equivalent to S^* in Fig 2B) is structurally weak, but it can recruit Polo—via phosphorylated S-S(P)/T(P) motifs (Alvarez-Rodrigo *et al*, 2019)—and also Cnn (Conduit *et al*, 2014b) to form the more stable $\bar{S}$ scaffold depicted in Fig 2B. The Polo recruited by Spd-2 is activated and can phosphorylate Cnn, allowing Cnn to assemble into a strong macromolecular scaffold (C^* in Fig 2B) (Conduit *et al*, 2014a; Feng *et al*, 2017). Cnn itself cannot recruit more Spd-2 or Polo, but it stabilises the expanding Spd-2 scaffold, so allowing Spd-2 to accumulate around the mother centriole (iii) (Conduit *et al*, 2014b).

Figure EV3. Model predictions are relatively robust to changes in parameter values.

Graphs show the computed output of Models 1 and 2 when each of the 13 reaction rate parameters is either doubled or halved (as indicated above each graph). The qualitative behaviour of the model is consistent in all cases, demonstrating the model's robustness in the parameter regime considered.

 © 2022 The Authors

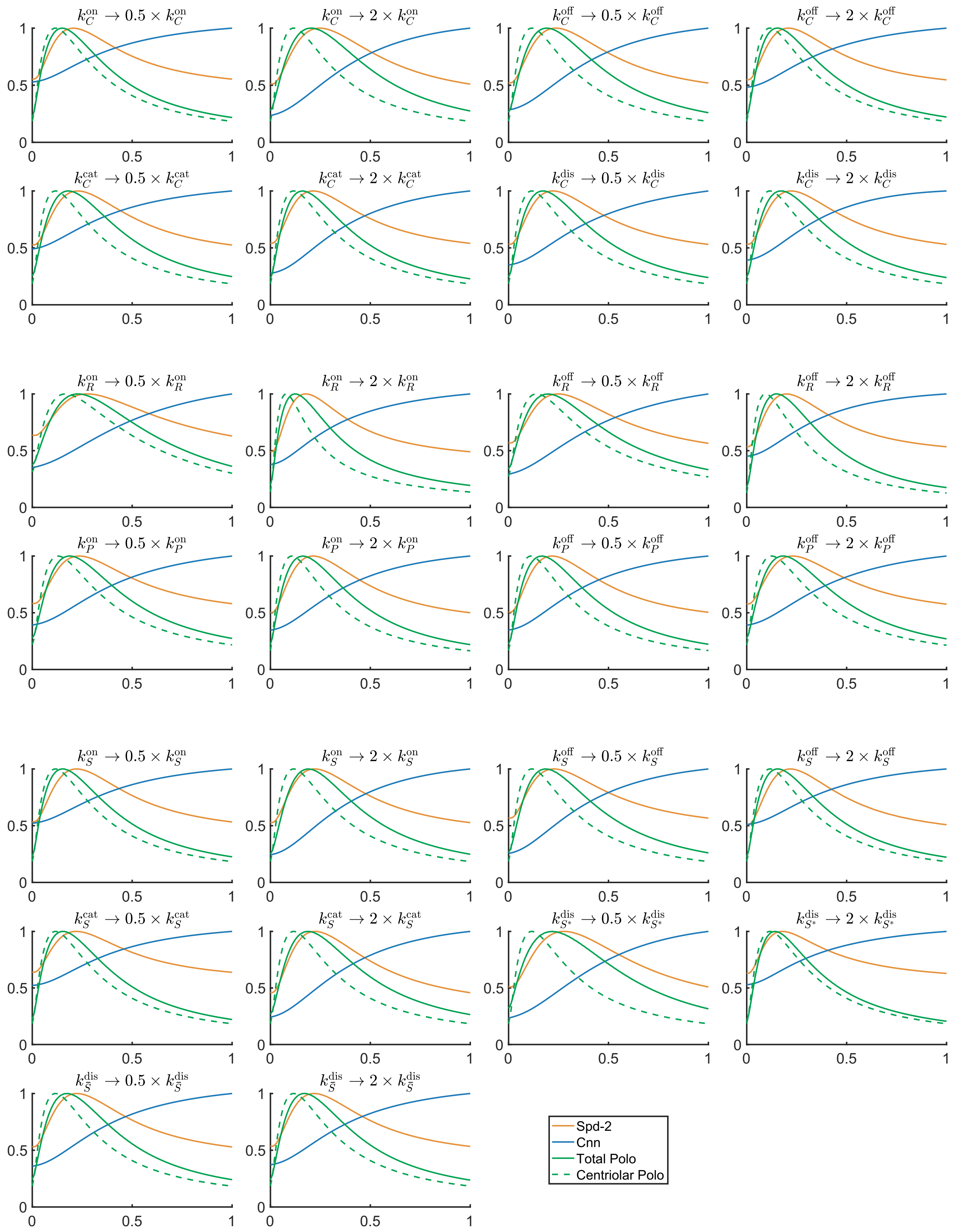
$k_C^{\mathrm{on}} \to 0.5 \times k_C^{\mathrm{on}}$
$k_C^{\mathrm{on}} \to 2 \times k_C^{\mathrm{on}}$
$k_C^{\mathrm{off}} \to 0.5 \times k_C^{\mathrm{off}}$
$k_C^{\mathrm{off}} \to 2 \times k_C^{\mathrm{off}}$
$k_C^{\mathrm{cat}} \to 0.5 \times k_C^{\mathrm{cat}}$
$k_C^{\mathrm{cat}} \to 2 \times k_C^{\mathrm{cat}}$
$k_C^{\mathrm{dis}} \to 0.5 \times k_C^{\mathrm{dis}}$
$k_C^{\mathrm{dis}} \to 2 \times k_C^{\mathrm{dis}}$
$k_R^{\mathrm{on}} \to 0.5 \times k_R^{\mathrm{on}}$
$k_R^{\mathrm{on}} \to 2 \times k_R^{\mathrm{on}}$
$k_R^{\mathrm{off}} \to 0.5 \times k_R^{\mathrm{off}}$
$k_R^{\mathrm{off}} \to 2 \times k_R^{\mathrm{off}}$
$k_P^{\mathrm{on}} \to 0.5 \times k_P^{\mathrm{on}}$
$k_P^{\mathrm{on}} \to 2 \times k_P^{\mathrm{on}}$
$k_P^{\mathrm{off}} \to 0.5 \times k_P^{\mathrm{off}}$
$k_P^{\mathrm{off}} \to 2 \times k_P^{\mathrm{off}}$
$k_S^{\mathrm{on}} \to 0.5 \times k_S^{\mathrm{on}}$
$k_S^{\mathrm{on}} \to 2 \times k_S^{\mathrm{on}}$
$k_S^{\mathrm{off}} \to 0.5 \times k_S^{\mathrm{off}}$
$k_S^{\mathrm{off}} \to 2 \times k_S^{\mathrm{off}}$
$k_S^{\mathrm{cat}} \to 0.5 \times k_S^{\mathrm{cat}}$
$k_S^{\mathrm{cat}} \to 2 \times k_S^{\mathrm{cat}}$
$k_{S^*}^{\mathrm{dis}} \to 0.5 \times k_{S^*}^{\mathrm{dis}}$
$k_{S^*}^{\mathrm{dis}} \to 2 \times k_{S^*}^{\mathrm{dis}}$
$k_{\bar{S}}^{\mathrm{dis}} \to 0.5 \times k_{\bar{S}}^{\mathrm{dis}}$
$k_{\bar{S}}^{\mathrm{dis}} \to 2 \times k_{\bar{S}}^{\mathrm{dis}}$
Spd-2
Cnn
Total Polo
Centriolar Polo

Figure EV3.

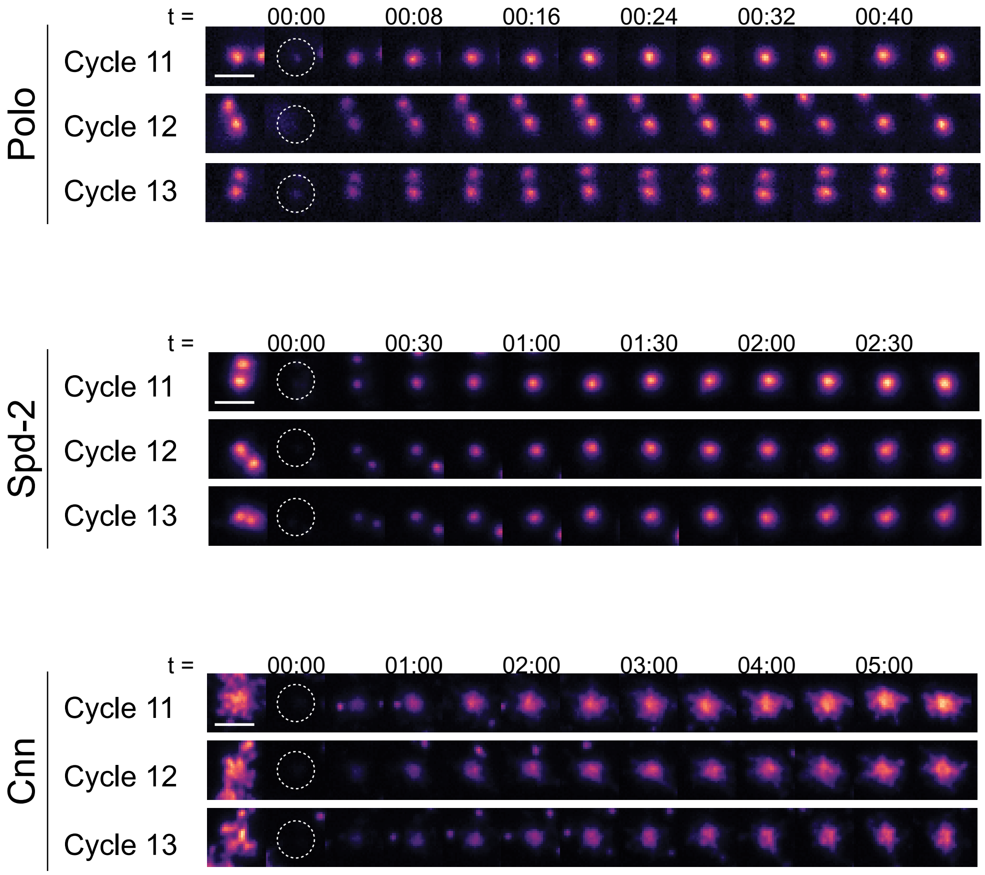

Figure EV4. Monitoring rates of centrosomal fluorescence recovery of photobleached PCM scaffold components.

Micrographs show examples of centrosomes that were fluorescently-labelled with either Polo-GFP, Spd-2-NG or NG-Cnn, photobleached at $t = 0$, and then monitored for the subsequent recovery of fluorescence at the start of either nuclear cycle 11, 12 or 13. Time (min:s) is indicated above selected images (note the different time scales used for each fusion protein). Scale bar = 2 μm.

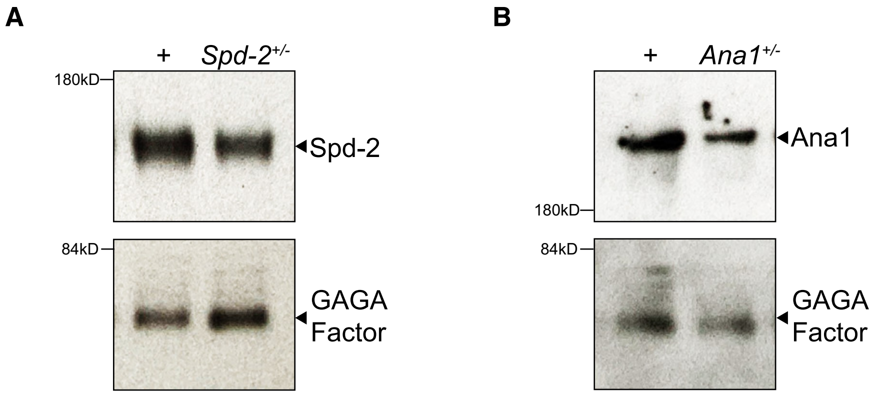

Figure EV5. Spd-2 and Ana1 appear to be expressed at lower levels in embryos laid by their respective heterozygous mutant mothers.

A, B Western blots show the levels of Spd-2 (A) or Ana1 (B) in embryos laid by either WT (+), or heterozygous (+/–) *Spd-2* or *Ana1* mutant females. Blots represent an example from two technical repeats. These blots were also probed with anti-GAGA factor antibodies as a loading control (Raff *et al*, 1994). The brightness and contrast were adjusted for optimal display.

 © 2022 The Authors
