# Supplementary material for: Centrioles generate a local pulse of Polo/PLK1 activity to initiate mitotic centrosome assembly
Source: EMBO J. 2022 May 3;41(11):e110891. doi: 10.15252/embj.2022110891 (PMC9156973; doi:10.15252/embj.2022110891)
Supplement: Supplementary file 3 [file EMBJ-41-e110891-s001.pdf]

THE EMBO JOURNAL

Centrioles generate a local pulse of Polo/PLK1 activity to initiate mitotic centrosome assembly

Siu-Shing Wong, Zachary M. Wilmott, Saroj Saurya, Ines Alvarez-Rodrigo, Felix Y. Zhou, Kwai-Yin Chau, Alain Goriely & Jordan W. Raff.
DOI: 10.15252/embj.2022110891

Corresponding author(s): Alain Goriely & Jordan W. Raff.

Review Timeline:

Transfer from Review Commons:	9th Feb 22
Editorial Decision:	4th Mar 22
Revision Received:	25th Mar 22
Accepted:	8th Apr 22

Editor: Hartmut Vodermaier

Transaction Report: This manuscript was transferred to The EMBO Journal following peer review at Review Commons.

(Note: With the exception of the correction of typographical or spelling errors that could be a source of ambiguity, letters and reports are not edited. Depending on transfer agreements, referee reports obtained elsewhere may or may not be included in this compilation. Referee reports are anonymous unless the Referee chooses to sign their reports.)

1st Editorial Decision 4th Mar 2022

Thank you for submitting your revised Review Commons manuscript for consideration by The EMBO Journal. In light of the positive original comments and the interest of the subject of the study, I decided to treat it similar to a regular revision, sending it directly to the original referees 1 and 2 for assessing your responses to the various specific points they had raised during review of the preprint. I am happy to say that both referees are overall satisfied with how their concerns had been addressed, and that pending incorporation of a few minor issues noted by referee 1, we would like to accept the study for publication.

I am therefore returning the study to you for a final round of minor revision, during which I would invite you to now also incorporate the following editorial and journal-format-specific points:

Referee #1:

Wong et al. build a mathematical model to explain the rapid assembly and disassembly of centrosomes in Drosophila embryos. Their model predicts how Polo kinase localization should change in response to changing levels of centriole docking receptors (e.g., Ana1) and scaffolding components (e.g., Spd-2). These predictions match the in vivo experiments and explain localization patterns of Polo that are not immediately intuitive. They conclude that a series of feedback mechanisms involving docking sites, stabilizing co-scaffolds, and phosphatases ensure that Polo kinase localization peaks early in mitosis and then drops rapidly.

The in vivo experiments are well controlled and quantified appropriately. The mathematical model appears effective and is robust, as it holds up after tweaking the parameters. The model makes many assumptions (e.g., relative rates of biochemical reactions; that Polo may cause its own dissociation from the Ana1 receptor). These assumptions may not be all true, but the model, on the whole, seems to have solid predictive power. One could request additional verification experiments (as is the case with any new experimental system), but I think that would be beyond the scope of this current paper.

Overall, this paper is novel and represents a significant advance in the centrosome field. I support its publication in the EMBO Journal. There are only a few minors concerns:

1. The authors claim that levels of Spd-2 and Ana1 are halved in Figure 6B and 6D. Is that actually the case? Could the authors provide a reference or a western blot to show that the protein levels are indeed halved? I say this because dosage compensation is extremely strong in the C. elegans germline; for example, protein levels can remain constant regardless if the worms has 1-4 copies of the gene.
2. Typo. Page 13, first paragraph. "We believe that Spd2-S16T-mCherry effects . . ." It should be "affects"
3. Typo, Figure S3. "Inavtive Polo"

4. Typo, Page 52, last paragraph. The wrong Table is referenced.

5. Referee #2:

I am satisfied with the changes introduced in the revised manuscript. In particular, the robustness analysis of the prediction (Fig 7) reinforces the computational conclusions.
Hence, I support the publication of the manuscript in the revised form.

Thank you for sending us the Reviewer's comments on the resubmission of our paper. Needless to say we are delighted with their positive responses and with your decision to accept the manuscript, pending our addressing the few remaining issues, which we have now done, as described below.

Reviewer 1 requested a western blot to show that when we halve the genetic dosage of *Spd-2* or *Ana1* in embryos, there is a corresponding reduction in protein levels in the embryo. We now provide such blots in a new Supplementary Figure (EV5). We have also corrected the typographical errors that the Reviewer spotted.

As you requested, we have also adjusted the manuscript to conform to the EMBO Journal formatting guidelines.

1st Revision - Editorial Decision 8th Apr 2022

Thank you for submitting your final revised manuscript for our consideration. I am pleased to inform you that we have now accepted it for publication in The EMBO Journal.

EMBO Press Author Checklist

Corresponding Author Name: Jordan W. Raff	
Journal Submitted to: EMBO Journal	
Manuscript Number: EMBOJ-2022-110891	

USEFUL LINKS FOR COMPLETING THIS FORM
[The EMBO Journal - Author Guidelines](#)
[EMBO Reports - Author Guidelines](#)
[Molecular Systems Biology - Author Guidelines](#)
[EMBO Molecular Medicine - Author Guidelines](#)

Reporting Checklist for Life Science Articles (updated January 2022)
This checklist is adapted from Materials Design Analysis Reporting (MDAR) Checklist for Authors. MDAR establishes a minimum set of requirements in transparent reporting in the life sciences (see Statement of Task: 10.31222/osf.io/9sm4x). Please follow the journal's guidelines in preparing your manuscript.
Please note that a copy of this checklist will be published alongside your article.

Abridged guidelines for figures
1. Data
The data shown in figures should satisfy the following conditions:
- the data were obtained and processed according to the field's best practice and are presented to reflect the results of the experiments in an accurate and unbiased manner.
- ideally, figure panels should include only measurements that are directly comparable to each other and obtained with the same assay.
- plots include clearly labeled error bars for independent experiments and sample sizes. Unless justified, error bars should not be shown for technical replicates.
- if n<5, the individual data points from each experiment should be plotted. Any statistical test employed should be justified.
- Source Data should be included to report the data underlying figures according to the guidelines set out in the authorship guidelines on Data Presentation.

2. Captions
Each figure caption should contain the following information, for each panel where they are relevant:
- a specification of the experimental system investigated (eg cell line, species name).
- the assay(s) and method(s) used to carry out the reported observations and measurements.
- an explicit mention of the biological and chemical entity(ies) that are being measured.
- an explicit mention of the biological and chemical entity(ies) that are altered/varied/perturbed in a controlled manner.
- the exact sample size (n) for each experimental group/condition, given as a number, not a range;
- a description of the sample collection allowing the reader to understand whether the samples represent technical or biological replicates (including how many animals, litters, cultures, etc.).
- a statement of how many times the experiment shown was independently replicated in the laboratory.
- definitions of statistical methods and measures:
  - common tests, such as t-test (please specify whether paired vs. unpaired), simple χ2 tests, Wilcoxon and Mann-Whitney tests, can be unambiguously identified by name only, but more complex techniques should be described in the methods section;
  - are tests one-sided or two-sided?
  - are there adjustments for multiple comparisons?
  - exact statistical test results, e.g., P values = x but not P values < x;
  - definition of 'center values' as median or average;
  - definition of error bars as s.d. or s.e.m.

Please complete ALL of the questions below.
Select "Not Applicable" only when the requested information is not relevant for your study.

Materials

Newly Created Materials	Information included in the manuscript?	In which section is the information available? (Reagents and Tools Table, Materials and Methods, Figures, Data Availability Section)
New materials and reagents need to be available; do any restrictions apply?	Yes	Data Availability

Antibodies	Information included in the manuscript?	In which section is the information available? (Reagents and Tools Table, Materials and Methods, Figures, Data Availability Section)
For **antibodies** provide the following information:<br>- Commercial antibodies: RRID (if possible) or supplier name, catalogue number and or/clone number<br>- Non-commercial: RRID or citation	Yes	Materials and methods

DNA and RNA sequences	Information included in the manuscript?	In which section is the information available? (Reagents and Tools Table, Materials and Methods, Figures, Data Availability Section)
**Short novel DNA or RNA including primers, probes**: provide the sequences.	Not Applicable	

Cell materials	Information included in the manuscript?	In which section is the information available? (Reagents and Tools Table, Materials and Methods, Figures, Data Availability Section)
**Cell lines:** Provide species information, strain. Provide accession number in repository **OR** supplier name, catalog number, clone number, and/**OR** RRID.	Not Applicable	
**Primary cultures:** Provide species, strain, sex of origin, genetic modification status.	Not Applicable	
Report if the cell lines were recently **authenticated** (e.g., by STR profiling) and tested for mycoplasma contamination.	Not Applicable	

Experimental animals	Information included in the manuscript?	In which section is the information available? (Reagents and Tools Table, Materials and Methods, Figures, Data Availability Section)
**Laboratory animals or Model organisms:** Provide species, strain, sex, age, genetic modification status. Provide accession number in repository **OR** supplier name, catalog number, clone number, **OR** RRID.	Yes	Materials and methods
**Animal observed in or captured from the field:** Provide species, sex, and age where possible.	Not Applicable	
Please detail **housing and husbandry conditions**.	Yes	Materials and methods

Plants and microbes	Information included in the manuscript?	In which section is the information available? (Reagents and Tools Table, Materials and Methods, Figures, Data Availability Section)
**Plants:** provide species and strain, ecotype and cultivar where relevant, unique accession number if available, and source (including location for collected wild specimens).	Not Applicable	
**Microbes:** provide species and strain, unique accession number if available, and source.	Not Applicable	

Human research participants	Information included in the manuscript?	In which section is the information available? (Reagents and Tools Table, Materials and Methods, Figures, Data Availability Section)
If collected and within the bounds of privacy constraints report on age, sex and gender or ethnicity for all study participants.	Not Applicable	

Core facilities	Information included in the manuscript?	In which section is the information available? (Reagents and Tools Table, Materials and Methods, Figures, Data Availability Section)
If your work benefited from core facilities, was their service mentioned in the acknowledgments section?	Yes	Acknowledgement

Design

Study protocol	Information included in the manuscript?	In which section is the information available? (Reagents and Tools Table, Materials and Methods, Figures, Data Availability Section)
If study protocol has been **pre-registered, provide DOI in the manuscript**. For clinical trials, provide the trial registration number **OR** cite DOI.	Not Applicable	
Report the **clinical trial registration number** (at ClinicalTrials.gov or equivalent), where applicable.	Not Applicable	

Laboratory protocol	Information included in the manuscript?	In which section is the information available? (Reagents and Tools Table, Materials and Methods, Figures, Data Availability Section)
Provide DOI OR other citation details if **external detailed step-by-step protocols** are available.	Not Applicable	

Experimental study design and statistics	Information included in the manuscript?	In which section is the information available? (Reagents and Tools Table, Materials and Methods, Figures, Data Availability Section)

Include a statement about **sample size** estimate even if no statistical methods were used.	Yes	Figures
Were any steps taken to minimize the effects of subjective bias when allocating animals/samples to treatment (e.g. **randomization procedure**)? If yes, have they been described?	Not Applicable	
Include a statement about **blinding** even if no blinding was done.	Not Applicable	
Describe **inclusion/exclusion criteria** if samples or animals were excluded from the analysis. Were the criteria pre-established?<br><br>If sample or data points were omitted from analysis, report if this was due to attrition or intentional exclusion and provide justification.	Yes	Figures, Results
For every figure, are **statistical tests** justified as appropriate? Do the data meet the assumptions of the tests (e.g., normal distribution)? Describe any methods used to assess it. Is there an estimate of variation within each group of data? Is the variance similar between the groups that are being statistically compared?	Yes	Figures

Sample definition and in-laboratory replication	Information included in the manuscript?	In which section is the information available?<br>(Reagents and Tools Table, Materials and Methods, Figures, Data Availability Section)
In the figure legends: state number of times the experiment was **replicated** in laboratory.	Yes	
In the figure legends: define whether data describe **technical or biological replicates**.	Yes	

Ethics

Ethics	Information included in the manuscript?	In which section is the information available?<br>(Reagents and Tools Table, Materials and Methods, Figures, Data Availability Section)
Studies involving **human participants**: State details of **authority granting ethics approval** (IRB or equivalent committee(s), provide reference number for approval.	Not Applicable	
Studies involving **human participants**: Include a statement confirming that **informed consent** was obtained from all subjects and that the experiments conformed to the principles set out in the WMA Declaration of Helsinki and the Department of Health and Human Services Belmont Report.	Not Applicable	
Studies involving **human participants:** For publication of **patient photos**, include a statement confirming that consent to publish was obtained.	Not Applicable	
Studies involving experimental **animals**: State details of **authority granting ethics approval** (IRB or equivalent committee(s), provide reference number for approval. Include a statement of compliance with ethical regulations.	Not Applicable	
Studies involving **specimen and field samples:** State if relevant **permits** obtained, provide details of authority approving study; if none were required, explain why.	Not Applicable	

Dual Use Research of Concern (DURC)	Information included in the manuscript?	In which section is the information available?<br>(Reagents and Tools Table, Materials and Methods, Figures, Data Availability Section)
Could your study fall under dual use research restrictions? Please check biosecurity documents and list of **select agents and toxins** (CDC): https://www.selectagents.gov/sat/list.htm	Not Applicable	
If you used a select agent, is the security level of the lab appropriate and reported in the manuscript?	Not Applicable	
If a study is subject to dual use research of concern regulations, is the name of the **authority granting approval and reference number** for the regulatory approval provided in the manuscript?	Not Applicable	

Reporting

The MDAR framework recommends adoption of discipline-specific guidelines, established and endorsed through community initiatives. Journals have their own policy about requiring specific guidelines and recommendations to complement MDAR.

Adherence to community standards	Information included in the manuscript?	In which section is the information available?<br>(Reagents and Tools Table, Materials and Methods, Figures, Data Availability Section)
State if relevant guidelines or checklists (e.g., **ICMJE, MIBBI, ARRIVE, PRISMA**) have been followed or provided.	Not Applicable	
For **tumor marker prognostic studies,** we recommend that you follow the **REMARK** reporting guidelines (see link list at top right). See author guidelines, under 'Reporting Guidelines'. Please confirm you have followed these guidelines.	Not Applicable	
For **phase II and III randomized controlled trials**, please refer to the **CONSORT** flow diagram (see link list at top right) and submit the CONSORT checklist (see link list at top right) with your submission. See author guidelines, under 'Reporting Guidelines'. Please confirm you have submitted this list.	Not Applicable	

Data Availability

Data availability	Information included in the manuscript?	In which section is the information available?<br>(Reagents and Tools Table, Materials and Methods, Figures, Data Availability Section)
Have **primary datasets** been deposited according to the journal's guidelines (see 'Data Deposition' section) and the respective accession numbers provided in the Data Availability Section?	Not Applicable	
Were **human clinical and genomic datasets** deposited in a public access-controlled repository in accordance to ethical obligations to the patients and to the applicable consent agreement?	Not Applicable	
Are **computational models** that are central and integral to a study available without restrictions in a machine-readable form? Were the relevant accession numbers or links provided?	Not Applicable	
If publicly available data were reused, provide the respective **data citations in the reference list**.	Not Applicable	
